# The Position of "The Hospital"

**Published:** 1895-04-06

**Authors:** 


### The Position of "The Hospital."

In commencing a new volume of The Hospital the Editor thinks it well to address a few words to his readers and his contributors.

In its inception The Hospital was principally concerned with hospital administration, hospital finance, and hospital construction, but, like everything which has life and power of survival, whether in biology or in journalism, it has accommodated itself to its environment. Now the environment of The Hospital is its *clientèle*, which includes all who are, in the broad sense of the term, interested in hospitals—medical staff and house surgeons, secretaries and committeemen, matrons, sisters, and nurses, architects and sanitary authorities, and in addition all that large mass of general medical practitioners who find that they best keep themselves abreast of modern progress in medical science by keeping themselves aware of what is going on in the hospitals, from which they know that knowledge springs.

It follows that the contents of The Hospital must be more varied in its character than is the case with some journals which are taken by a more strictly limited, and, as some would say, more strictly professional class, and have, therefore, to limit their choice of topics to those relating to the daily duties of one class and not to the common interests of all who take part in the work of our hospitals and medical institutions. The common link in the readers of The Hospital is care for the sick and helpless, and whether this takes the form of medical science, treatment, or therapeutics, hospital construction, institutional management, or general sanitation, each department is interested in the work of others in the same field, however different from their own special branch it may appear to be. The press, the profession, and the public recognise these facts, and the time cannot be far distant when they will be grasped by the manufacturers, who have so far failed to realise the exceptional advantages they can secure in the columns of The Hospital.

To cater for so varied a *clientèle* is a matter of no small anxiety, and would have been impossible if the Editor had not taken care to call to his aid experts in the various departments of knowledge dealt with. Especially in regard to the more purely medical portion he has secured the assistance of a large number of professional collaborators, by whose energy he is able, week by week, to present to his readers a survey of what is going on in the medical world, which he is assured, by impartial critics, is interesting, exhaustive, and accurate, and well suited to the circumstances of those for whose benefit it is written. The Editor does not hesitate to attribute much of the success of The Hospital to the system on which it is managed, which differs notably from that which obtains in regard to most other English medical journals. The Hospital is absolutely independent. The Editor is uncontrolled by any committee, the policy of the paper is unfettered by deference to any influential subscribers. Every article is paid for, and the Editor is under obligation to no one. On the other hand, every article which is not thought likely to be useful is sent back to the contributor, who is thus relieved from the suspense of not knowing what is going to be done with his idea. At the same time the Editor does not sit in judgment on his contributors; rejection implies no blame, but merely that the contribution is not what he wants. It is hoped that this system will be found much more satisfactory to writers than the pigeon-holing plan so commonly adopted. That it is successful will be granted by any who read the clinics and other contributions which appear in The Hospital, written as they are by men connected with the leading hospitals and medical schools in London and throughout the country.

The Hospital, then, occupies this position—it pays for all it prints and is cap-in-hand to no one; the public, on the other hand, pays for what it reads, for there is no free distribution. Yet The Hospital is to be found in every hospital of importance throughout the British Empire. The Editor can thus feel the pulse of that medical and official world which constitutes the public to whom he appeals, and he is able to tell at once by the circulation whether or no The Hospital fills a void. That it does fill a void, and that it does meet the requirements of its readers, its growing circulation assures him. Every week the sales increase, evidence is forthcoming that members of the medical profession take it in in preference to any other journal, and it is now admitted to be the most widely read and popular of all the medical papers.

